# Real‐world treatment patterns and quality of life among patients with locally advanced or metastatic urothelial carcinoma living in Saudi Arabia, South Korea, Taiwan, and Turkey

**DOI:** 10.1111/iju.15497

**Published:** 2024-05-24

**Authors:** Li‐Jen Cheng, Janet Kim, Apurba Mukherjee, Neil Milloy, Mia Unsworth, Daniel Ng

**Affiliations:** ^1^ Medical Affairs Astellas Pharma Singapore Pte, Ltd. Singapore; ^2^ Biostatistics Astellas Pharma Global Development Northbrook Illinois USA; ^3^ Adelphi Real World Bollington UK

**Keywords:** metastatic, quality of life, treatment patterns, urothelial carcinoma

## Abstract

**Objectives:**

To evaluate demographic and clinical characteristics, treatment patterns, and quality of life in patients with locally advanced or metastatic urothelial carcinoma in Asia.

**Methods:**

Data were drawn from the Adelphi Real World Metastatic Urothelial Carcinoma Disease Specific Programme™, a cross‐sectional survey of medical oncologists/urologists and their adult patients in Saudi Arabia, South Korea, Taiwan, and Turkey. Exploratory patient‐reported outcomes included the EQ‐5D visual analog scale, European Organisation for Research and Treatment of Cancer Quality of Life of Patient Questionnaire global health, and Brief Pain Inventory. Analyses were descriptive.

**Results:**

Overall, 175 physicians reported data for 988 patients. Mean (standard deviation) patient age was 66.3 (10.8) years, 77% were men, and 82% had bladder tumors at diagnosis. Of patients receiving first‐ (*n* = 988), second‐ (*n* = 290), and third‐line (*n* = 87) treatments, 81%, 35%, and 59% received chemotherapy, respectively, and 17%, 63%, and 34% received programmed cell death protein 1/ligand 1 inhibitors, respectively. Patient‐reported (*n* = 319) mean (standard deviation) EQ‐5D visual analog scale score was 51.8 (15.6), European Organisation for Research and Treatment of Cancer Quality of Life of Patient Questionnaire global health status score was 44.6 (19.9), and Brief Pain Inventory score was 6.5 (1.9; *n* = 315).

**Conclusion:**

The most common first‐ and second‐line treatments for locally advanced or metastatic urothelial carcinoma were chemotherapy and programmed cell death protein 1/ligand inhibitors, respectively. At third line, 10% of patients received best supportive care alone, underscoring an unmet need for effective third‐line treatment options. Patients in all regions reported quality‐of‐life impairment.

**Abbreviations & Acronyms**
1L: first line
2L: second line
3L: third line
BSC: best supportive care
DSP: Disease Specific Programme
ECOG PS: Eastern Cooperative Oncology Group performance status
EORTC: European Organisation for Research and Treatment of Cancer
max: maximum
min: minimum
mUC: metastatic urothelial carcinoma
OS: overall survival
PD‐1/L1: programmed cell death protein 1/ligand 1
QL2: global health status
QLQ‐C30: Quality of Life of Patient Questionnaire
QOL: quality of life
SD: standard deviation
UC: urothelial carcinoma
VAS: visual analog scale

## INTRODUCTION

Urothelial carcinoma (UC), the most common type of bladder cancer,^1^, ^2^, ^3^ can be located in the lower and/or upper urinary tract, with approximately 8% of patients with UC presenting with local disease recurrence.^3^, ^4^, ^5^ One‐year prevalence rates of bladder cancer in 2020 were 733 per 100 000 in Saudi Arabia, 4262 per 100 000 in South Korea, and 9562 per 100 000 people in Turkey.^6^ In Taiwan, bladder cancer had a reported incidence rate of 10 per 100 000 in 2020.^7^

Standard‐of‐care first‐line treatment for locally advanced or metastatic UC (la/mUC) is platinum‐based chemotherapy (eg, cisplatin and carboplatin).^8^ Regimens, including platinum‐based treatment, have demonstrated a median overall survival (OS) of approximately 9 to 15 months,^4^, ^8^, ^9^, ^10^, ^11^ with lower median OS (4–7 months) after relapse following first‐line therapy.^8^, ^12^ Switch maintenance therapy with a checkpoint inhibitor may help delay disease progression after first‐line treatment and is recommended by European guidelines.^4^, ^13^ Second‐line treatment includes programmed cell death protein 1/ligand 1 (PD‐1/L1) inhibitors and taxanes. If mUC progresses with checkpoint inhibitor treatment, few treatment options exist.^4^, ^8^ Subsequent treatment includes enfortumab vedotin, a Nectin‐4–directed antibody–drug conjugate,^4^, ^14^ and targeted agents such as erdafitinib (a fibroblast growth factor receptor inhibitor used to treat tumors harboring susceptible *FGFR2/3* alterations) or chemotherapy.^4^ Recent studies also suggest that first‐line treatment with nivolumab plus gemcitabine‐cisplatin or with enfortumab vedotin plus pembrolizumab may improve outcomes versus gemcitabine plus platinum‐based chemotherapy.^15^, ^16^ However, novel regimens and some therapies typically used in second‐ or later‐line are not yet universally available.^4^, ^17^

Treatments for la/mUC used in second‐ or later‐line have not been widely studied,^17^ and data are lacking for real‐world treatment patterns and quality‐of‐life (QoL) among patients with mUC in Saudi Arabia, South Korea, Taiwan, and Turkey.^18^, ^19^, ^20^, ^21^ This study assessed demographic and clinical characteristics, treatment patterns, and patient‐reported QoL outcomes in patients with la/mUC treated in these regions.

## METHODS

### Data sources and study design

Data were from the Adelphi Real World mUC Disease Specific Programme™ (DSP; Bollington, UK), a cross‐sectional survey with retrospective data collection of medical oncologists and urologists and their adult patients with mUC in Saudi Arabia, South Korea, Taiwan, and Turkey from December 2021 to March 2022. Physicians completed patient record forms for patients with mUC, reporting demographics, clinical characteristics, tests/assessments, and treatments received. These patients were invited to complete a self‐completion questionnaire detailing their overall QoL and disease‐specific QoL, as well as the impact of la/mUC on work, activity, and functioning via the EQ‐5D VAS and utility score, European Organisation for Research and Treatment of Cancer (EORTC) Quality‐of‐Life of Patient Questionnaire (QLQ‐C30) QL2, QoL, and overall health scores, Work Productivity and Activity Index (WPAI), and Brief Pain Inventory. The DSP methodology has been previously validated and published.^22^, ^23^

### Data collection

Data were collected from December 15, 2021, to March 31, 2022, excluding incomplete surveys. Patient surveys were not collected in South Korea due to data protection/ethics laws.

### Physician and patient eligibility criteria

Physician sampling was pragmatic (based on physician availability); patient sampling was pseudorandom based on order of consultation. Physicians completed patient record forms for four to eight consecutive consulting eligible patients to mitigate selection bias. Physicians were eligible if they were personally responsible for the care of patients with la/mUC, spent at least 50% of their time in patient care, and saw at least five patients per month.

Patients were eligible if they were aged 18 years or older, had a physician‐confirmed diagnosis of la/mUC, and were not involved in a clinical trial at data collection.

### Endpoints

Primary endpoints were patient demographics and clinical characteristics, therapies received in first‐, second‐, and third‐line settings for la/mUC, and proportion of patients treated by line of therapy or who remained untreated after the second‐line setting. Data about current and prior treatment lines were collected retrospectively. Secondary endpoints were types of health care professional involved in patient diagnosis, care, and initiation of therapy; surgeries received; history of hospital stay; and biomarker/genetic tests conducted. Switch maintenance therapy was defined as receiving treatment with an agent with a different mode of action after completion of induction chemotherapy in patients whose tumors had not progressed.

Patient‐reported outcomes were exploratory endpoints. Assessments included the EQ‐5D visual analog scale (VAS) and utility score, global health status (QL2) score from the EORTC QLQ‐C30, EORTC QoL and overall health scores, Work Productivity and Activity Index, and Brief Pain Inventory scores.

EQ‐5D VAS scores range from 0 (worst imaginable health) to 100 (best imaginable health), and EQ‐5D utility scores range from less than 0 (where 0 indicates a health state equivalent to death) to 1 (full health).^24^ EORTC QLQ‐C30 scores range from 0 (low functioning) to 100 (high functioning), with EORTC QoL scores and overall health scores ranging from 1 (very poor) to 7 (excellent) over the last 7 days.^25^ Work Productivity and Activity Index scores range from 0 to 10 across four domains (absenteeism, presenteeism, overall work performance, and non‐work activities), with a score of 10 indicating the most impairment.^26^ Brief Pain Inventory scores range from 0 (no pain and no interference in daily functioning) to 10 (worst imaginable pain or pain that completely interferes with daily functioning).^27^

### Statistical analyses

Analyses were descriptive. Patient cohorts were defined by line of treatment at the time of the survey and stratified by region. Exploratory subgroup analysis assessed treatments received stratified by physician specialty. All data analysis was conducted in UNICOM Intelligence Reporter version 7.5.1.19 (UNICOM Systems, Mission Hills, CA).

## RESULTS

### Physician and patient samples

**FIGURE 1:**
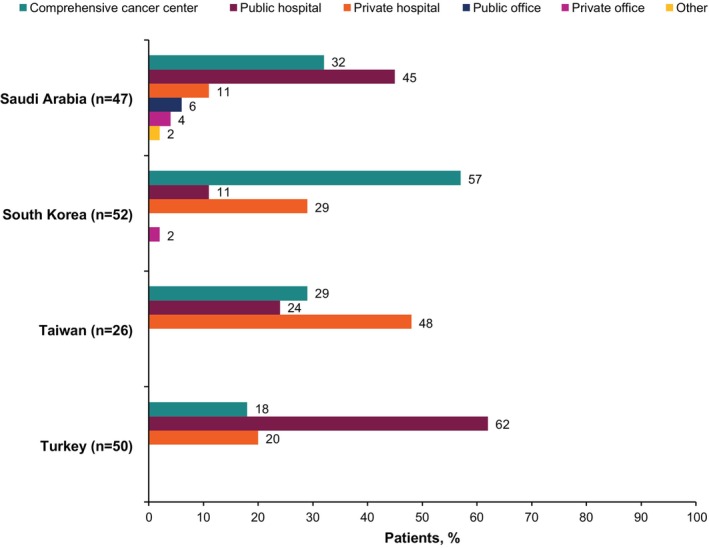
Patients receiving outpatient or ambulatory care as seen by practice setting and by location.

Data were provided by 175 physicians (Saudi Arabia, *n* = 47; South Korea, *n* = 52; Taiwan, *n* = 26; Turkey, *n* = 50) for 988 patients with la/mUC (Saudi Arabia, *n* = 240; South Korea, *n* = 298; Taiwan, *n* = 150; Turkey, *n* = 300; Table 1). Overall, 99 physicians were medical oncologists (Saudi Arabia, *n* = 29; South Korea, *n* = 18; Taiwan, *n* = 17; Turkey, *n* = 35) and 76 were urologists (Saudi Arabia, *n* = 18; South Korea, *n* = 34; Taiwan, *n* = 9; Turkey, *n* = 15). Most physicians practiced in public hospitals or comprehensive cancer centers (Figure 1). The mean number of patient record forms per physician was 6. Patient self‐completion surveys were available for 319 patients (Saudi Arabia, *n* = 89; Taiwan, *n* = 24; Turkey, *n* = 206).

**TABLE 1:** Patient demographics and clinical characteristics.

Parameter	Total *N* = 988	Saudi Arabia *n* = 240	South Korea *n* = 298	Taiwan *n* = 150	Turkey *n* = 300
Age, mean (SD) years	66.3 (10.8)	54.9 (7.9)	68.5 (9.4)	68.6 (10.7)	72.2 (6.4)
Male, *n* (%)	759 (77)	152 (63)	239 (80)	88 (59)	280 (93)
Risk factor, *n* (%)					
Genetic condition (eg, Lynch syndrome)	19 (2)	0 (0)	0 (0)	0 (0)	19 (6)
Smoking	677 (69)	130 (54)	191 (64)	60 (40)	296 (99)
Occupational exposure	30 (3)	0 (0)	2 (1)	2 (1)	26 (9)
Water arsenic exposure	1 (<1)	0 (0)	0 (0)	1 (1)	0 (0)
Treatment received (phenacetin, cyclophosphamide, chlornaphazine)	1 (<1)	0 (0)	1 (<1)	0 (0)	0 (0)
Radiation exposure	5 (1)	0 (0)	4 (1)	1 (1)	0 (0)
Other^a^	5 (1)	0 (0)	1 (<1)	4 (3)	0 (0)
None	299 (30)	110 (46)	103 (35)	83 (55)	3 (1)
Smoking status, *n* (%)					
Current	149 (15)	93 (39)	22 (7)	18 (12)	16 (5)
History of smoking	523 (53)	34 (14)	169 (57)	40 (27)	280 (93)
No history	283 (29)	112 (47)	85 (29)	82 (55)	4 (1)
Unknown	33 (3)	1 (<1)	22 (7)	10 (7)	0 (0)
Family history of UC, *n* (%)					
Yes	39 (4)	7 (3)	7 (2)	0 (0)	25 (8)
No	762 (77)	233 (97)	239 (80)	118 (79)	172 (57)
Unknown	187 (19)	0 (0)	52 (17)	32 (21)	103 (34)
Tumor location, *n* (%)					
Ureter	78 (8)	0 (0)	53 (18)	22 (15)	3 (1)
Renal pelvis	97 (10)	1 (<1)	48 (16)	44 (29)	4 (1)
Bladder	809 (82)	239 (100)	195 (65)	82 (55)	293 (98)
Urethra	4 (<1)	0 (0)	2 (1)	2 (1)	0 (0)
Metastatic site at data collection, *n* (%)					
Lymph node	519 (53)	33 (14)	211 (71)	100 (67)	175 (58)
Lung	283 (29)	96 (40)	116 (39)	49 (33)	22 (7)
Liver	268 (27)	153 (64)	36 (12)	24 (16)	55 (18)
Bone	212 (21)	71 (30)	73 (24)	32 (21)	36 (12)
Perinephric tissue	28 (3)	0 (0)	11 (4)	12 (8)	5 (2)
Other	24 (2)	0 (0)	18 (6)	6 (4)	0 (0)
Pancreas	21 (2)	0 (0)	0 (0)	0 (0)	21 (7)
Ureter	21 (2)	0 (0)	11 (4)	5 (3)	5 (2)
Adrenal gland	14 (1)	4 (2)	5 (2)	3 (2)	2 (1)
Urethra	9 (1)	0 (0)	6 (2)	3 (2)	0 (0)
Renal pelvis	7 (1)	0 (0)	1 (<1)	1 (1)	5 (2)
Bladder	7 (1)	0 (0)	4 (1)	1 (1)	2 (1)
Brain	5 (1)	4 (2)	1 (<1)	0 (0)	0 (0)
Major vein	5 (1)	0 (0)	2 (1)	3 (2)	0 (0)
Time since UC diagnosis,^b^ mean (SD), months	34.0 (32.8)	24.1 (17.8)	42.2 (36.7)	40.9 (43.9)	19.3 (13.1)
Time since locally advanced or metastatic UC diagnosis, mean (SD), months	11.4 (13.6)	15.3 (9.9)	15.4 (19.9)	10.0 (11.1)	5.0 (3.7)
ECOG PS, *n* (%)					
0	178 (18)	9 (4)	125 (42)	43 (29)	1 (<1)
1	308 (31)	85 (35)	127 (43)	76 (51)	20 (7)
2	331 (34)	96 (40)	38 (13)	22 (15)	175 (58)
3	152 (15)	37 (15)	3 (1)	8 (5)	104 (35)
4	10 (1)	9 (4)	0 (0)	1 (1)	0 (0)
Unknown/unanswered	9 (1)	4 (2)	5 (2)	0 (0)	0 (0)

Abbreviations: ECOG PS, Eastern Cooperative Oncology Group performance status; SD, standard deviation; UC, urothelial carcinoma.

^a^ Collected as an open text response and was not coded due to low base.

^b^ Total: *n* = 377; Turkey, *n* = 25; Saudi Arabia: *n* = 118; South Korea: *n* = 136; Taiwan: *n* = 58.

### Patient characteristics

Patient demographics and clinical characteristics are reported in Table 1. Although almost one‐third of patients overall reported no risk factors, smoking was the most common risk factor, especially among Turkish patients. The bladder was the most common initial tumor location at UC diagnosis, with high rates in Turkey and Saudi Arabia. Other tumor locations commonly included the renal pelvis, ureter, and urethra. At initial UC diagnosis, over two‐thirds of patients had tumor–node–metastasis stage IVA M0, IVA M1, or IVB. Approximately one‐half of patients had an Eastern Cooperative Oncology Group performance status of 0 (fully active) to 1 (restrictions on physically strenuous activity).

### Treatment patterns

Overall (*n* = 988), almost three‐quarters of patients were eligible for cisplatin, ranging from approximately half of patients in Taiwan to almost all patients in Saudi Arabia. Main criteria for determining cisplatin eligibility were renal function status (by creatinine clearance) and Eastern Cooperative Oncology Group performance status (Table S1). The proportions of patients receiving first‐, second‐, and third‐line therapy are detailed in Table S2. Proportions of patients receiving first‐line therapy ranged from just over half of patients in South Korea to almost all patients in Turkey (Table 2). In patients initially diagnosed with non‐mUC, chemotherapy was the most common neoadjuvant and adjuvant therapy across locations (Table 2).

**TABLE 2:** Treatment patterns by location, stratified by diagnosis of locally advanced or metastatic urothelial carcinoma.

Parameter, *n* (%)	Total *N* = 346	Saudi Arabia *n* = 118	South Korea *n* = 142	Taiwan *n* = 61	Turkey *n* = 25
Prior nonsurgical treatment related to UC (before locally advanced or metastatic UC diagnosis)					
Yes, active drug (not associated with surgery)	104 (30)	57 (48)	23 (16)	11 (18)	13 (52)
Yes, radiotherapy (not associated with surgery)	19 (5)	7 (6)	7 (5)	4 (7)	1 (4)
No active drug/radiotherapy	212 (61)	50 (42)	107 (75)	44 (72)	11 (44)
Unknown	19 (5)	5 (4)	10 (7)	4 (7)	0 (0)
Neoadjuvant treatment received before diagnosis	*n* = 98	*n* = 72	*n* = 15	*n* = 6	*n* = 5
Chemotherapy	96 (98)	71 (99)	14 (93)	6 (100)	5 (100)
PD‐1/L1 inhibitor	1 (1)	0 (0)	0 (0)	1 (17)	0 (0)
Other^a^	31 (32)	26 (36)	4 (27)	1 (17)	0 (0)
Adjuvant treatment received before diagnosis	*n* = 107	*n* = 34	*n* = 43	*n* = 19	*n* = 11
Chemotherapy	94 (88)	33 (97)	38 (88)	13 (68)	10 (91)
PD‐1/L1 inhibitor	1 (1)	0 (0)	0 (0)	1 (5)	0 (0)
Other^a^	39 (36)	16 (47)	13 (30)	9 (47)	1 (9)
Treatment not associated with surgery received before diagnosis	*n* = 115	*n* = 63	*n* = 25	*n* = 13	*n* = 14
Chemotherapy	93 (81)	53 (84)	20 (80)	8 (62)	12 (86)
PD‐1/L1 inhibitor	9 (8)	6 (10)	1 (4)	2 (15)	0 (0)
Other^a^	25 (22)	7 (11)	10 (40)	5 (38)	3 (21)

Abbreviations: PD‐1/L1, programmed cell death protein 1/ligand 1; UC, urothelial carcinoma.

^a^ Includes targeted therapy, radiotherapy, Bacillus Calmette–Guérin vaccine, and other therapies.

#### First‐line treatment patterns

**FIGURE 2:**
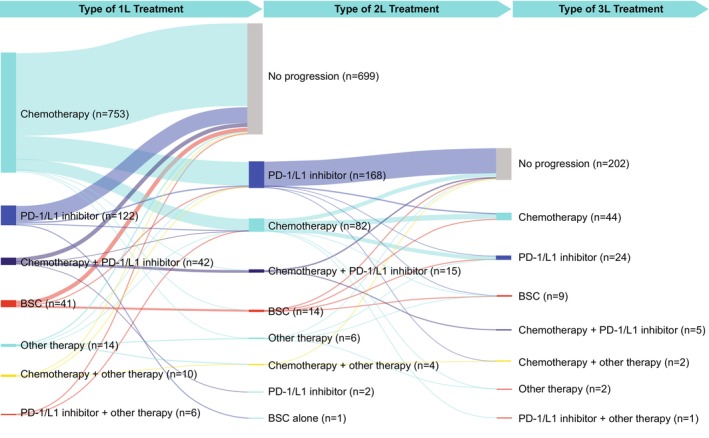
Treatment type received at first‐, second‐, and third‐line settings overall. 1L, first‐line; 2L, second‐line; 3L, third‐line; BSC, best supportive care; PD‐1/L1, programmed cell death protein 1/ligand 1.

**FIGURE 3:**
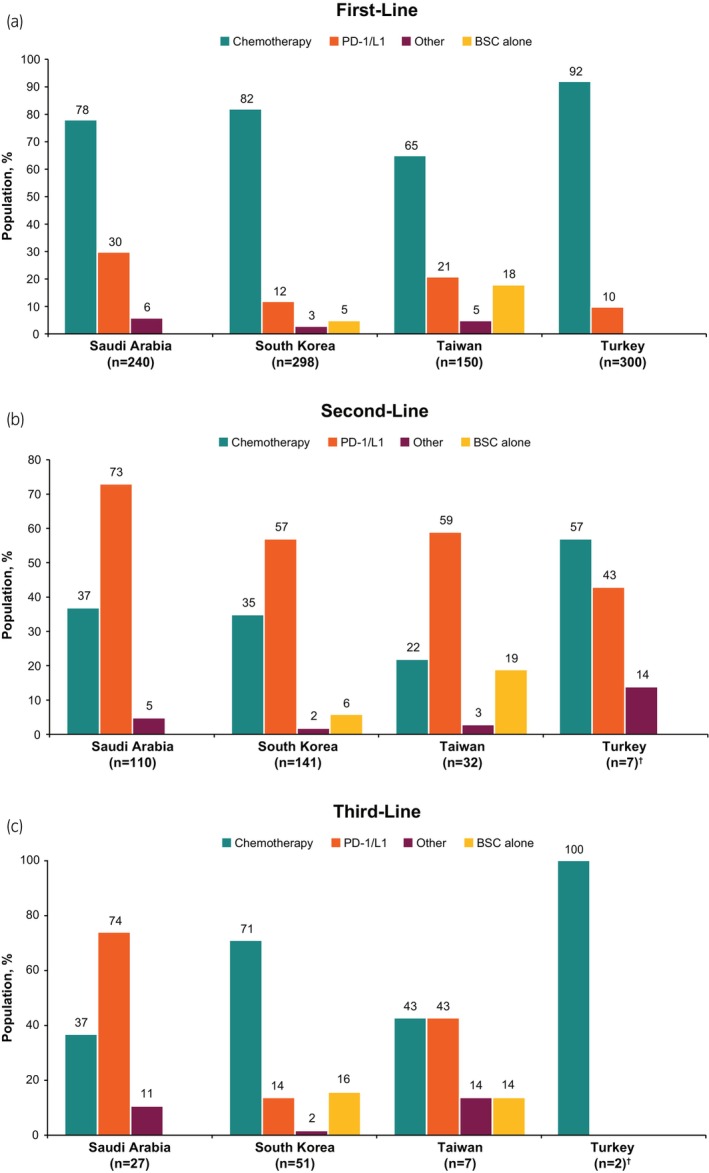
Treatment type received at (a) first‐, (b) second‐, and (c) third‐line settings by location. Total percentages may exceed 100% because responses were not mutually exclusive. BSC, best supportive care; PD‐1/L1, programmed cell death protein 1/ligand 1. ^†^Data insufficient to interpret the result from Turkey in second‐ (*n* = 7) and third‐line (*n* = 2) settings.

At first‐line (*n* = 988), most patients received chemotherapy (mostly gemcitabine, *n* = 727 [74%], followed by cisplatin, *n* = 588 [60%], and carboplatin, *n* = 175 [18%]) and under one‐fifth received PD‐1/L1 inhibitors (including avelumab, *n* = 35 [4%]); a small percentage of patients received best supportive care alone (Figure 2; Table S2). Proportions of patients receiving first‐line chemotherapy ranged from almost two‐thirds in Taiwan to almost all patients in Turkey (Figure 3a). Use of PD‐1/L1 inhibitors ranged from one‐tenth in Turkey to almost one‐third in Saudi Arabia (Figure 3b).

Few patients (*n* = 25) received switch maintenance therapy (gemcitabine, *n* = 7 [28%]; atezolizumab, *n* = 6 [24%]), with very few (*n* = 2 [8%]) receiving avelumab (Table S2).

#### Second‐line and treatment patterns

Of 290 patients receiving second‐line treatment, more than one‐third received chemotherapy (gemcitabine, *n* = 78 [27%]; cisplatin, *n* = 77 [27%]), nearly two‐thirds received a PD‐1/L1 inhibitor (atezolizumab, *n* = 77 [27%]; pembrolizumab, *n* = 63 [22%]), and a low percentage of patients received best supportive care alone or received another type of treatment (Figure 2; Table S2). Use of PD‐1/L1 inhibitors ranged from approximately two‐fifths in Turkey to almost three‐quarters in Saudi Arabia (Figure 3b). In South Korea and Taiwan, more than half of patients received second‐line PD‐1/L1 inhibitors (Figure 3b). Data were insufficient to interpret use of second‐line treatments in Turkey.

#### Third‐line treatment patterns

Of the 87 patients receiving third‐line treatment, more than half received chemotherapy (cisplatin, *n* = 40 [46%]; gemcitabine, *n* = 33 [38%]), approximately one‐third received a PD‐1/L1 inhibitor (pembrolizumab, *n* = 16 [18%]; nivolumab, *n* = 10 [11%]), one‐tenth received best supportive care alone, and a smaller percentage received another type of treatment (Figure 2; Table S2). Less than half of patients received third‐line PD‐1/L1 inhibitors in South Korea and Taiwan (Figure 3c). Data were insufficient to interpret use of third‐line treatments in Turkey.

#### Treatment duration

In patients whose treatment periods were known, mean (SD) treatment duration for first‐, second‐, and third‐line treatments were 5.7 (7.1) months (*n* = 335), 4.9 (5.0) months (*n* = 125), and 4.3 (7.7) months (*n* = 29), respectively (Table S3).

### Health care resource utilization and costs

Overall, most patients received a diagnosis and initiated treatment from a medical oncologist or a urologist in this sample, with a higher proportion of urologists in South Korea and Taiwan (Table 3). Medical history, physical examination findings, complete blood count, and alanine aminotransferase and aspartate aminotransferase levels were mostly used to aid in the diagnosis of la/mUC. A total of 18% of patients underwent surgery for la/mUC, primarily transurethral resection of the bladder tumor and radical nephroureterectomy.

**TABLE 3:** Health care resource utilization by location.

Parameter, *n* (%)	Total	Saudi Arabia	South Korea	Taiwan	Turkey
Health care professional responsible for initial diagnosis	*n* = 988	*n* = 240	*n* = 298	*n* = 150	*n* = 300
Primary care physician/Family physician	22 (2)	11 (5)	10 (3)	0 (0)	1 (<1)
Medical oncologist	372 (38)	130 (54)	55 (18)	33 (22)	154 (51)
Urologist	585 (59)	99 (41)	227 (76)	114 (76)	145 (48)
Surgical oncologist	5 (1)	0 (0)	5 (2)	0 (0)	0 (0)
Radiologist/Radiation oncologist	2 (<1)	0 (0)	1 (<1)	1 (1)	0 (0)
Other	2 (<1)	0 (0)	0 (0)	2 (1)	0 (0)
Surgery received for UC at nonmetastatic stage	*n* = 293	*n* = 91	*n* = 134	*n* = 49	*n* = 19
Transurethral resection of prostate	3 (1)	2 (2)	1 (1)	0 (0)	0 (0)
Transurethral resection of bladder tumor	172 (59)	76 (84)	68 (51)	24 (49)	4 (21)
Transurethral resection of urethral tumor	6 (2)	0 (0)	0 (0)	0 (0)	6 (32)
Radical cystectomy	53 (18)	10 (11)	38 (28)	3 (6)	2 (11)
Partial cystectomy	13 (4)	0 (0)	5 (4)	4 (8)	4 (21)
Radical nephroureterectomy	75 (26)	3 (3)	53 (40)	17 (35)	2 (11)
Urethrectomy	2 (1)	0 (0)	0 (0)	2 (4)	0 (0)
Regional lymphadenectomy	11 (4)	0 (0)	6 (4)	5 (10)	0 (0)
Pelvic exenteration	1 (<1)	0 (0)	1 (1)	0 (0)	0 (0)
Other surgery	4 (1)	0 (0)	2 (1)	1 (2)	1 (5)
Surgery received for locally advanced or metastatic UC	*n* = 179	*n* = 32	*n* = 109	*n* = 37	*n* = 1
Transurethral resection of prostate	3 (2)	0 (0)	2 (2)	1 (3)	0 (0)
Transurethral resection of bladder tumor	110 (61)	31 (97)	56 (51)	23 (62)	0 (0)
Transurethral resection of urethral tumor	3 (2)	0 (0)	1 (1)	1 (3)	1 (100)
Radical cystectomy	18 (10)	0 (0)	15 (14)	3 (8)	0 (0)
Partial cystectomy	7 (4)	0 (0)	4 (4)	3 (8)	0 (0)
Radical nephroureterectomy	41 (23)	0 (0)	35 (32)	6 (16)	0 (0)
Urethrectomy	3 (2)	0 (0)	3 (3)	0 (0)	0 (0)
Regional lymphadenectomy	20 (11)	0 (0)	13 (12)	7 (19)	0 (0)
Pelvic exenteration	2 (1)	0 (0)	0 (0)	2 (5)	0 (0)
Other surgery	10 (6)	1 (3)	7 (6)	2 (5)	0 (0)

Abbreviation: UC, urothelial carcinoma.

**FIGURE 4:**
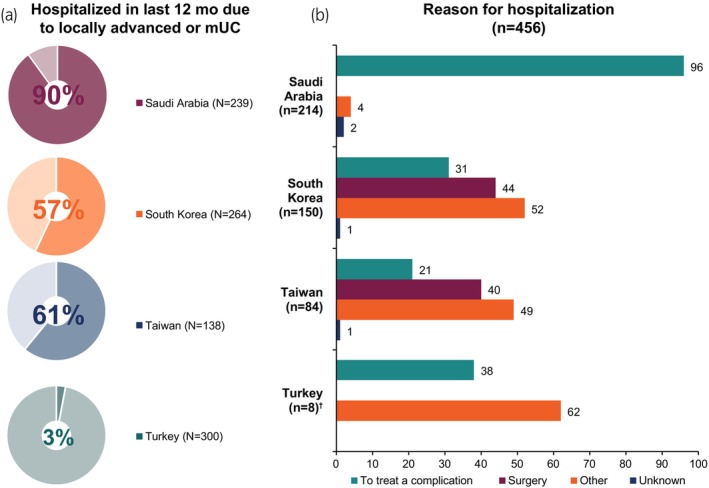
History of hospital stay by location among patients with locally advanced or mUC, (a) Hospitalizations in the past 12 months due to locally advanced or mUC. (b) Reasons for that hospital stay. mUC, metastatic urothelial carcinoma. ^†^Data insufficient to interpret the result from Turkey (*n* = 8).

Overall, almost half of patients had at least one hospital stay in the past 12 months for la/mUC, ranging from 3% in Turkey to 90% in Saudi Arabia (Figure 4a). Mean (SD) overall number of hospital stays due to la/mUC in the past year was 1.1 (1.9). Delineated by country, these rates were 1.8 (2.9) in Taiwan, 1.6 (2.4) in South Korea, 1.5 (0.9) in Saudi Arabia, and 0 (0.2) in Turkey. Primary reasons for hospital stays were treatment of a complication of la/mUC in Saudi Arabia and for surgery for la/mUC in South Korea and Taiwan (Figure 4b).

### Biomarker testing

Overall, biomarker/tumor testing occurred in 55% (*n* = 545) of patients and ranged from more than one‐tenth of patients in Turkey to all patients in Saudi Arabia (Table 4). The most frequent tests reported were PD‐1/L1 expression status, particularly in Saudi Arabia.

**TABLE 4:** Biomarker analysis by location.

Biomarker/Genetic test, *n* (%)	Total *N* = 988	Saudi Arabia *n* = 240	South Korea *n* = 298	Taiwan *n* = 150	Turkey *n* = 300
PD‐1	99 (10)	15 (6)	33 (11)	36 (24)	15 (5)
PD‐L1	478 (48)	225 (94)	140 (47)	76 (51)	37 (12)
*EGFR*	14 (1)	0 (0)	13 (4)	1 (1)	0 (0)
*Her‐2/neu*	12 (1)	0 (0)	12 (4)	0 (0)	0 (0)
*mTOR*	5 (1)	0 (0)	5 (2)	0 (0)	0 (0)
*PI3K*	14 (1)	0 (0)	13 (4)	1 (1)	0 (0)
*c‐MET*	10 (1)	0 (0)	10 (3)	0 (0)	0 (0)
*AXL*	3 (<1)	0 (0)	3 (1)	0 (0)	0 (0)
*IDO*	1 (<1)	0 (0)	1 (<1)	0 (0)	0 (0)
*FGFR2*	89 (9)	72 (30)	15 (5)	2 (1)	0 (0)
*FGFR3*	80 (8)	60 (25)	19 (6)	1 (1)	0 (0)
*VEGF*	14 (1)	0 (0)	14 (5)	0 (0)	0 (0)
Other	2 (<1)	0 (0)	2 (1)	0 (0)	0 (0)
Missing	443 (45)	0 (0)	133 (45)	47 (31)	263 (88)

Abbreviations: *AXL*, AXL receptor tyrosine kinase; *c‐MET*, mesenchymal‐epithelial transition factor; *EGFR*, epidermal growth factor receptor; *FGFR*, fibroblast growth factor receptor; *Her‐2/neu*, human epidermal growth factor receptor 2; *IDO*, indoleamine 2, 3‐dioxygenase 1; *mTOR*, mammalian target of rapamycin; *PI3K*, phosphoinositide 3‐kinases; PD‐1, programmed cell death protein 1; PD‐L1, programmed cell death ligand 1; *VEGF*, vascular endothelial growth factor receptor.

### Patient‐reported outcomes

**FIGURE 5:**
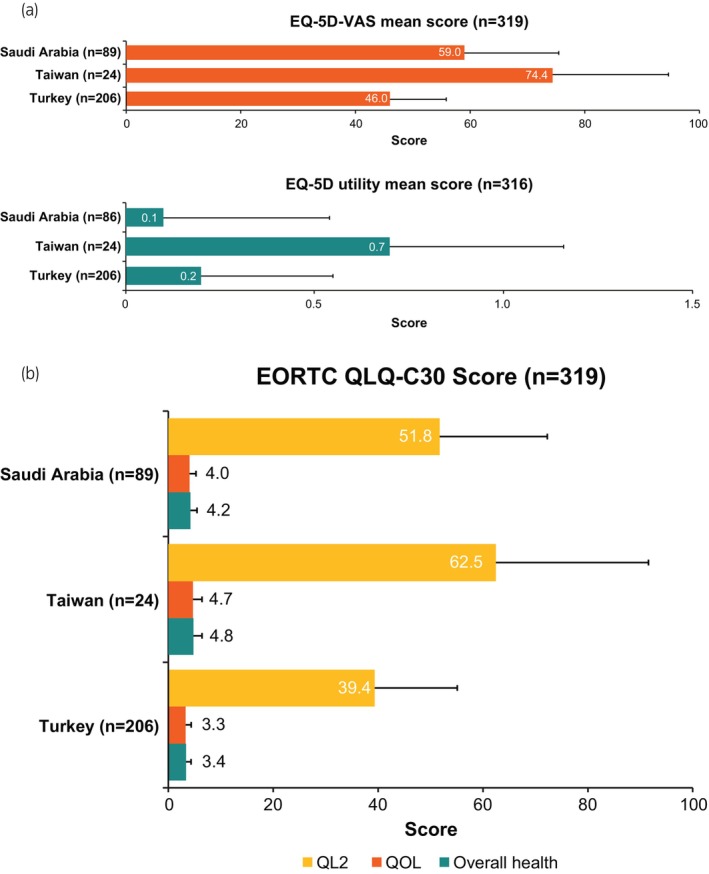
Patient‐reported (a) EQ‐5D and (b) EORTC QLQ‐C30 scores by location. Error bars represent standard deviation. EQ‐5D VAS scores range from 0 (worst health the patient can imagine) to 100 (best health the patient can imagine). EORTC overall health and QoL scores are measured 1 (very poor) to 7 (excellent) over the last 7 days. QL2 is measured 0–100, with a higher score indicating a better QoL. EORTC QLQ‐C30, European Organisation for Research and Treatment of Cancer Quality of Life of Patient Questionnaire; QL2, global health status; QoL, quality of life; VAS, visual analog scale.

Overall (*n* = 319) mean (SD) EQ‐5D VAS score was 51.8 (15.6), and mean (SD) EQ‐5D score among 316 patients was 0.2 (0.4); scores by country are presented in Figure 5a.

Overall mean (SD) EORTC QLQ‐C30 QL2 score was 44.6 (19.9), lower than previously published normative data for the general population (mean [SD]: 71.2 [22.4]).^28^ Mean (SD) EORTC QLQ‐C30 QoL and overall health scores were 3.6 (1.3) and 3.7 (1.2), respectively; scores by country are presented in Figure 5b.

Mean (SD) effect of health conditions on ability to perform activities not related to work (WPAI) was 6.5 (2.0), and overall mean pain severity (worst pain on Brief Pain Inventory) was 6.5 (1.9; Tables S4 and S5).

## DISCUSSION

This study helped to address the knowledge gap about real‐world demographics, clinical characteristics, treatment patterns, and patient‐reported outcomes for adults with la/mUC in Saudi Arabia, South Korea, Taiwan, and Turkey. In agreement with real‐world findings in other regions, most patients had prior exposure to tobacco smoking,^19^, ^20^ an established risk factor for bladder cancer.^29^ Platinum‐based chemotherapies and PD‐1/L1 inhibitors were common first‐ and second‐line treatments.^19^, ^21^ However, a lower proportion of patients were receiving third‐line treatment; 59% of patients with available data after second‐line treatment received third‐line chemotherapy and 10% received best supportive care alone. This suggests an unmet need for third‐line treatment options. Substantial resource use and QoL impairment were also observed in the la/mUC patient population in these regions.

These findings should be interpreted in the context of local and international treatment guidelines. Cisplatin or carboplatin were frequent first‐line treatments among patients whose data were included in this study. This finding is a consequence of natural fallouts from the database and is also related to the high rate of cisplatin eligibility in Saudi Arabia. Inhibitors of PD‐1/L1 were the second most common first‐line treatment class for la/mUC in this analysis, in agreement with guidelines that recommend first‐line PD‐1/L1 inhibitors for patients ineligible for platinum‐based chemotherapy with a positive PD‐L1 biomarker test (European Society for Medical Oncology^4^) and in patients ineligible for platinum‐based therapy regardless of PD‐L1 expression (National Comprehensive Cancer Network^4^, ^8^). The proportion of patients receiving chemotherapy as first‐line treatment in the present study was generally consistent with other real‐world studies conducted in Canada,^21^ Spain,^30^ and United States of America.^19^

Physician prescribing PD‐1/L1 inhibitors was influenced by reimbursement and availability of specific treatments in the regions studied in addition to treatment guidelines. For example, the proportion of patients receiving first‐line PD‐1/L1 inhibitors in this study was lower than a real‐world study conducted in Spain,^30^ possibly due to variations in reimbursement between regions, as first‐line checkpoint inhibitors are reimbursed only for cisplatin‐ineligible patients in Taiwan and are not reimbursed in South Korea and Turkey. Although European guidelines recommend maintenance treatment for patients with la/mUC whose disease has not progressed on first‐line platinum‐based chemotherapy,^4^ only 25 patients in the present study received maintenance therapy, likely due to lack of reimbursement for avelumab maintenance therapy in these regions. Also, PD‐1/L1 inhibitors were the most common second‐line treatments in the present study, in agreement with treatment recommendations.^4^, ^8^ At second‐line treatment, proportions of patients receiving PD‐1/L1 inhibitors were generally similar in real‐world studies conducted in Canada^21^ and Spain.^30^ This finding reflects favorable reimbursement status of second‐line PD‐1/L1 inhibitors in the regions studied.

In this study, cisplatin was the main third‐line chemotherapy option, and 10% of patients with data for third‐line treatment received best supportive care alone. By contrast, rates of third‐line platinum‐based chemotherapy were lower in real‐world studies from Spain^30^ and the United States of America.^19^ Enfortumab vedotin and erdafitinib were not available in these regions during the study period. Together, these findings suggest availability of alternative treatments for use in second‐ or later‐line may be more limited in the regions studied than in other parts of the world at the time of data collection. The lack of treatment options challenges the effective management of mUC in emerging markets,^31^ and improving access to advanced treatments may help address disparities between regions.

This study found substantial health care resource utilization, including 48% of patients with at least one hospital stay in the past year, and QoL impairment among patients with la/mUC in locations where data on the impact of la/mUC on patients' lives are scarce. These findings were consistent with a Spanish real‐world study that also found a high rate of inpatient admission in patients with la/mUC.^30^ Real‐world studies conducted in the United States of America before the availability of most novel agents, found that patients who did not receive treatment after discontinuing first‐ or second‐line PD‐1/L1 inhibitor therapy had low overall survival, substantial resource use, economic burden, and QoL impairment.^32^, ^33^ Together, these findings highlight the importance of increasing access to effective later treatment lines, managing symptom burden, which can affect outcomes, and addressing QoL in patients with la/mUC.^34^

### Limitations

In the DSP, which is based on a pseudorandom sample of physicians or patients, physician participation was influenced by willingness to complete the survey; however, inclusion criteria governing physician selection were minimal. To minimize patient selection bias, physicians were asked to provide data for a consecutive series of eligible patients. Patient eligibility was based on the responding physician's judgment, not on a formalized diagnostic checklist; however, it is representative of physicians' real‐world classification of their patients.

The point‐in‐time survey design prevented conclusions about causal relationships; however, identification of significant associations was possible. Recall bias, a common limitation of surveys, might have affected responses of physicians and patients. However, physicians could refer to patient records while completing the form, thus minimizing recall bias. Data were collected at each patient's appointment to reduce the likelihood of recall bias when the physician's opinion was required.

Patients with la/mUC in this multiregional study generally received first‐line platinum‐based treatment and second‐line PD‐1/L1 inhibitor treatment. Options for third‐line treatment were limited, highlighting the need to better manage survival outcomes. Exploratory analyses also suggested QoL impairment in this population. These real‐world findings could help increase knowledge of la/mUC management in real‐world practice and highlight the importance of developing new third‐line treatment options.

## AUTHOR CONTRIBUTIONS

**Li‐Jen Cheng:** Writing—review & editing. **Janet Kim:** Writing—review & editing. **Apurba Mukherjee:** Writing—review & editing. **Neil Milloy:** Data curation; Writing—review & editing. **Mia Unsworth:** Data curation; Writing—review & editing. **Daniel Ng:** Writing—review & editing.

## CONFLICT OF INTEREST STATEMENT

Li‐Jen Cheng is an employee of Astellas Pharma Singapore Pte, Ltd. Janet Kim is an employee of Astellas Pharma, Inc. Apurba Mukherjee is an employee of Astellas Pharma Singapore Pte, Ltd. Neil Milloy is an employee of Adelphi Real World. Mia Unsworth is an employee of Adelphi Real World. Daniel Ng is an employee of Astellas Pharma Singapore Pte, Ltd.

## APPROVAL OF THE RESEARCH PROTOCOL BY AN INSTITUTIONAL REVIEWER BOARD

The survey was conducted according to European Pharmaceutical Marketing Research Association guidelines and no ethics committee approval was required. Survey materials were reviewed and approved by the Western Institutional Review Board (study number AG8913). Each survey was performed in full accordance with relevant legislation at the time of data collection, including the US Health Insurance Portability and Accountability Act 1996 and Health Information Technology for Economic and Clinical Health Act legislation. Committee of Pearl IRB, exemption number AG8913.

The Institutional review board reviewed the documents submitted for exemption determination in accordance with FDA 21 CFR 56.104 and DHHS 45 CFR 46.104 regulations and approved the request. The protocol 21‐ADRW‐128. Protocol mUC v1.0 011221 was determined by the institutional review board to be exempt according to FDA 21 CFR 56.104 and 45CFR46.104(b) (2): (2) Tests, Surveys, Interviews on 12/03/2021.

## INFORMED CONSENT

Informed consent was obtained from all patients.

## REGISTRY AND THE REGISTRATION NO. OF THE STUDY/TRIAL

Study/trial registry and registration number are not applicable.

## ANIMAL STUDIES

Animal Studies registry and registration number are not applicable.

## FUNDING INFORMATION

Data collection was undertaken by Adelphi Real World as part of an independent survey titled the Adelphi Real World mUC DSP. The DSP is a wholly owned Adelphi Real World product. Astellas Pharma was one of multiple subscribers to the DSP. Astellas Pharma did not influence the original survey through either contribution to the design of questionnaires or data collection. This data analysis was funded by Astellas Pharma, Inc. and Seagen Inc, which was acquired by Pfizer in Dec. 2023.

## Supporting information


Tables S1–S5.

## Data Availability

Researchers may request access to anonymized participant level data, trial level data, and protocols from Astellas sponsored clinical trials at www.clinicalstudydatarequest.com. For the Astellas criteria on data sharing see: https://clinicalstudydatarequest.com/Study‐Sponsors/Study‐Sponsors‐Astellas.aspx. Data collection was undertaken by Adelphi Real World as part of an independent survey entitled the Adelphi Metastatic Urothelial Cancer Disease Specific Programme™ (DSP). The DSP is a wholly owned Adelphi product; all methodology, materials, data, and data analysis that support the findings of this survey are the intellectual property of Adelphi Real World and are not publicly available. All requests for access should be addressed directly to Mia Unsworth at mia.unsworth@adelphigroup.com.
