# Supplementary material for: Real‐world treatment patterns and quality of life among patients with locally advanced or metastatic urothelial carcinoma living in Saudi Arabia, South Korea, Taiwan, and Turkey
Source: Int J Urol. 2024 May 24;31(8):933–43. doi: 10.1111/iju.15497 (PMC11524135; doi:10.1111/iju.15497)
Supplement: Supplementary file 1 — Tables S1–S5. [file IJU-31-933-s001.docx]

**SUPPLEMENTARY INFORMATION**

**Supplementary Table S1.** Criteria used by physicians to determine cisplatin eligibility by location

| Parameter, n (%) | Total  (N=689) | Saudi Arabia  (n=233) | South Korea  (n=208) | Taiwan  (n=77) | Turkey  (n=171) |
| --- | --- | --- | --- | --- | --- |
| PD-1/L1 status | 263 (38) | 203 (87) | 29 (14) | 22 (29) | 9 (5) |
| Impaired renal function^†^ | 597 (87) | 230 (99) | 173 (83) | 51 (66) | 143 (84) |
| Age | 260 (38) | 21 (9) | 53 (25) | 27 (35) | 159 (93) |
| ECOG PS | 546 (79) | 226 (97) | 162 (78) | 44 (57) | 114 (67) |
| Solitary kidney status | 253 (37) | 162 (70) | 33 (16) | 18 (23) | 40 (23) |
| Status of heart failure | 355 (52) | 224 (96) | 60 (29) | 26 (34) | 45 (26) |
| Status of hearing loss | 368 (53) | 217 (93) | 43 (21) | 18 (23) | 90 (53) |
| Status of peripheral neuropathy | 425 (62) | 223 (96) | 80 (38) | 38 (49) | 84 (49) |

*Note:* ^†^Assessed using rate of creatinine clearance. Abbreviations: ECOG PS, Eastern Cooperative Oncology Group performance status; PD-1/L1, programmed cell death protein 1/ligand 1.

**Supplementary Table S2.** Proportion of patients and treatment received at each line of therapy by location

| Therapy, n (%) | Total | Saudi Arabia | South Korea | Taiwan | Turkey |
| --- | --- | --- | --- | --- | --- |
| Line of therapy | N=988 | n=240 | n=298 | n=150 | n=300 |
| First | 698 (71) | 130 (54) | 157 (53) | 118 (79) | 293 (98) |
| Second | 203 (21) | 83 (35) | 90 (30) | 25 (17) | 5 (2) |
| Third or later | 87 (9) | 27 (11) | 51 (17) | 7 (5) | 2 (1) |
| First-line | N=988 | n=240 | n=298 | n=150 | n=300 |
| Chemotherapy | 805 (81) | 187 (78) | 245 (82) | 97 (65) | 276 (92) |
| PD-1/L1 | 170 (17) | 71 (30) | 37 (12) | 31 (21) | 31 (10) |
| Other^†^ | 30 (3) | 14 (6) | 8 (3) | 8 (5) | 0 (0) |
| Best supportive care alone | 41 (4) | 0 (0) | 14 (5) | 27 (18) | 0 (0) |
| Second-line | n=290 | n=110 | n=141 | n=32 | n=7 |
| Chemotherapy | 102 (35) | 41 (37) | 50 (35) | 7 (22) | 4 (57) |
| PD-1/L1 | 183 (63) | 80 (73) | 81 (57) | 19 (59) | 3 (43) |
| Other^‡^ | 11 (4) | 6 (5) | 3 (2) | 1 (3) | 1 (14) |
| Best supportive care alone | 14 (5) | 0 (0) | 8 (6) | 6 (19) | 0 (0) |
| Third-line | n=87 | n=27 | n=51 | n=7 | n=2 |
| Chemotherapy | 51 (59) | 10 (37) | 36 (71) | 3 (43) | 2 (100) |
| PD-1/L1 | 30 (34) | 20 (74) | 7 (14) | 3 (43) | 0 (0) |
| Other^§^ | 5 (6) | 3 (11) | 1 (2) | 1 (14) | 0 (0) |
| Best supportive care alone | 9 (10) | 0 (0) | 8 (16) | 1 (14) | 0 (0) |
| Switch maintenance therapy | n=25 | n=4 | n=19 | n=2 | — |
| Chemotherapy | 8 (32) | 2 (50) | 5 (26) | 1 (50) | — |
| PD-1/L1 | 11 (44) | 3 (75) | 8 (42) | 0 (0) | — |
| Best supportive care alone | 1 (4) | 0 (0) | 1 (5) | 0 (0) | — |

*Notes:* Total percentages may exceed 100%. ^†^Includes erdafitinib (14 [1%]), Bacillus Calmette–Guerin (5% [1%]), pegfilgrastim (3 [<1%]), and other (8% [1%]). ^‡^Includes erdafitinib (7 [2%]), Bacillus Calmette–Guerin (3 [1%]), and pegfilgrastim (1 [<1%]). ^§^Includes erdafitinib (3 [3%]), and other (2 [2%]). Abbreviations: PD-1/L1, programmed cell death protein 1/ligand 1.

**Supplementary Table S3.** Treatment duration by location

| Parameter | Total | Saudi Arabia | South Korea | Taiwan | Turkey |
| --- | --- | --- | --- | --- | --- |
| Duration of first-line treatment, months | n=355 | n=12 | n=196 | n=117 | n=10 |
| Mean (SD) | 5.7 (7.1) | 5.0 (2.4) | 5.6 (7.3) | 5.5 (6.6) | 11.0 (11.3) |
| Range (min, max) | 0–43 | 2–11 | 0–43 | 0–40 | 2–41 |
| Duration of second-line treatment, months | n=125 | n=4 | n=91 | n=26 | n=4 |
| Mean (SD) | 4.9 (5.0) | 3.5 (1.0) | 5.0 (5.3) | 5.0 (4.6) | 2.5 (2.4) |
| Range (min, max) | 0–26 | 2–4 | 0–26 | 1–23 | 0–5 |
| Duration of third-line treatment, months | n=29 | n=0 | n=24 | n=3 | n=2 |
| Mean (SD) | 4.3 (7.7) | 0 (0) | 4.4 (8.4) | 4.7 (4.0) | 2.5 (3.5) |
| Range (min, max) | 0–36 | 0–0 | 0–36 | 0–7 | 0–5 |

Abbreviations: max, maximum; min, minimum; SD, standard deviation.

**Supplementary Table S4.** Work productivity and activity impairment^†^ assessment by location

| Parameter | Total | Saudi Arabia | Taiwan | Turkey |
| --- | --- | --- | --- | --- |
| Employed at time of data collection | n=319 | n=89 | n=24 | n=206 |
| Yes, n (%) | 26 (8) | 21 (24) | 2 (8) | 3 (1) |
| Time missed from work in last seven days associated with health problems, hours | n=26 | n=21 | n=2 | n=3 |
| Mean (SD) | 13.7 (10.8) | 14.9 (11.4) | 3.5 (0.7) | 12.7 (6.4) |
| Range (min, max) | 0–40 | 0–40 | 3–4 | 8–20 |
| Time missed from work in last seven days due to other reason(s), hours | n=26 | n=21 | n=2 | n=3 |
| Mean (SD) | 4.5 (7.9) | 5.3 (8.6) | 0 (0) | 2.0 (2.0) |
| Range (min, max) | 0–24 | 0–24 | 0–0 | 0–4 |
| Time worked in the last seven days, hours | n=26 | n=21 | n=2 | n=3 |
| Mean (SD) | 23.7 (13.7) | 23.3 (14.9) | 33.0 (4.2) | 20.0 (0) |
| Range (min, max) | 0–45 | 0–45 | 30–36 | 20–20 |
| Effect^†^ of health problems on work productivity over last seven days | n=21 | n=18 | n=2 | n=1 |
| Mean (SD) | 5.7 (1.7) | 6.1 (1.3) | 2.0 (0) | 6.0 (0) |
| Range (min, max) | 2–9 | 4–9 | 2–2 | 6–6 |
| Effect^†^ of health problems on ability to perform daily activities, excluding work, over the last seven days | n=319 | n=89 | n=24 | n=206 |
| Mean (SD) | 6.5 (2.0) | 6.2 (1.7) | 4.5 (3.5) | 6.9 (1.6) |
| Range (min, max) | 0–10 | 1–9 | 0–9 | 1–10 |

*Note:* ^†^Scored 0–10, with 0 indicating no effect. Abbreviations: max, maximum; min, minimum; SD, standard deviation.

**Supplementary Table S5.** Brief pain inventory^†^ assessment by location

| Pain severity | Total | Saudi Arabia | Taiwan | Turkey |
| --- | --- | --- | --- | --- |
| Worst | n=315 | n=89 | n=20 | n=206 |
| Mean (SD) | 6.5 (1.9) | 5.7 (1.8) | 3.0 (2.7) | 7.3 (1.1) |
| Range (min, max) | 0–9 | 0–9 | 0–8 | 3–9 |
| Average | n=315 | n=89 | n=20 | n=206 |
| Mean (SD) | 5.6 (1.7) | 5.1 (1.7) | 1.9 (1.8) | 6.2 (1.0) |
| Range (min, max) | 0–8 | 0–8 | 0–5 | 2–8 |
| Interference | n=315 | n=89 | n=20 | n=206 |
| Mean (SD) | 5.7 (1.9) | 5.1 (1.7) | 1.9 (2.6) | 6.3 (1.2) |
| Range (min, max) | 0–9 | 0–9 | 0–7 | 3–9 |

*Note:* ^†^Scored 0–10, with 0 being no pain/no interference to 10 being worst imaginable pain or pain that completely interferes. Abbreviations: max, maximum; min, minimum; SD, standard deviation.
